# Genome-Wide Association Study and Candidate Gene Mining for First Pod Height in Soybean

**DOI:** 10.3390/plants15192937

**Published:** 2026-09-25

**Authors:** Xiuli Yue, Tengfei Liu, Chunhua Wu, Yandang Liu, Siyuan Chen, Kai Zhang, Jianbo He, Junyi Gai, Yan Li

**Affiliations:** State Key Laboratory of Crop Genetics & Germplasm Enhancement and Utilization, National Center for Soybean Improvement, Key Laboratory for Biology and Genetic Improvement of Soybean, Jiangsu Collaborative Innovation Center for Modern Crop Production, Nanjing Agricultural University, Nanjing 210095, China

**Keywords:** soybean, first pod height, GWAS, QTN, *GmAIR9*, haplotype

## Abstract

First pod height (FPH) is a critical agronomic trait in soybean because it directly affects mechanized harvesting efficiency and harvestable yield. Pods below the cutter-bar height are left uncollected, leading to direct seed loss. However, the genetic architecture underlying FPH remains largely underexplored. Here, we conducted a genome-wide association study (GWAS) on FPH data using 386 soybean accessions evaluated across three environments, together with best linear unbiased prediction (BLUP) values. Phenotypic analysis showed that cultivated soybean exhibited significantly greater FPH than wild accessions, consistent with selection for improved harvestability during domestication. Using two GWAS methods, RTM-GWAS and EMMAX, we identified seven stable (detected by the same method at least three times) quantitative trait nucleotides/loci (QTNs/QTLs) significantly associated with FPH. By integrating sequence variation, gene expression, and functional description, we identified *GmAIR9* (corresponding to *Glyma.09G084300/SoyZH13_09G078900*), which encodes a microtubule-associated protein preferentially expressed in stems, as a candidate gene for further investigation. Haplotype analysis of *GmAIR9* revealed that haplotype H3 was associated with low FPH and was detected exclusively in wild soybean accessions, whereas haplotype H1 was associated with increased FPH. Moreover, the frequency of H1 increased from 50.00% in wild soybeans to 71.03% in landraces and 83.47% in improved cultivars. These findings provide valuable genetic resources and promising candidate loci for future validation and breeding applications to optimize FPH and improve the suitability of soybean cultivars for mechanized harvesting.

## 1. Introduction

Soybean is one of the world’s most economically important leguminous crops and serves as a major source of vegetable oil and high-quality protein for human consumption and animal feed [1,2,3]. Over the past few decades, global demand for soybean has increased dramatically, necessitating continuous improvements in yield. First pod height (FPH), also referred to as bottom pod height, is an important agronomic trait for mechanized harvesting. FPH directly affects harvestable yield by determining the proportion of pods positioned above the cutter-bar height of combine harvesters. The cutter-bar height cannot be set too low; otherwise, physical damage to the equipment may occur due to stones or other debris on the soil surface. Therefore, if the FPH is insufficient, the lowest pods may be cut, damaged, or entirely missed during harvesting, leading to substantial yield losses [4,5,6]. Conversely, excessively high FPH may compromise plant stability and lodging resistance [6]. Using ninety soybean genotypes during 2008 and 2009, Ramteke et al. [7] reported a significant negative correlation between lodging degree and pre-lodging FPH (*r* = −0.224, *p* < 0.05). As mechanized harvesting is increasingly adopted in large-scale soybean production, harvest losses associated with excessively low FPH remain an important constraint on harvesting efficiency and harvestable yield. Consequently, developing soybean cultivars with an appropriate FPH to facilitate efficient mechanical harvesting has become an important objective of modern soybean breeding programs [8].

FPH is a typical quantitative trait controlled by multiple genes and influenced by environmental factors [8]. However, relatively few quantitative trait loci (QTLs) and genes underlying FPH have been reported in soybean. Jiang et al. [8] identified 11 major QTLs for FPH using 147 soybean recombinant inbred lines and subsequently identified eight candidate genes potentially involved in plant growth, and seven genes exhibited high expression levels in soybean stems. Wang et al. [9] identified a novel QTL, *qBPH-O-2*, associated with bottom pod height. Previous studies have demonstrated that FPH is influenced by both node number and internode length and is also correlated with plant height [6]. The SoyBase [10] database (https://www.soybase.org) and several genome-wide association studies (GWAS) have documented numerous QTLs and quantitative trait nucleotides (QTNs) associated with other plant architecture traits such as node number, internode length, and plant height in soybean [11,12,13,14]. However, the genetic loci and candidate genes underlying FPH remain largely underexplored [8,9], particularly in natural populations and across multiple environments. Notably, previous studies have primarily relied on cultivated soybean panels, leaving the allelic diversity present in wild soybean largely untapped for FPH dissection. In this study, we addressed these gaps by performing GWAS on a diverse population of 386 soybean accessions encompassing wild, landrace, and cultivated types, evaluated across three environments. This population enables us to capture a broader range of phenotypic and genetic variation, facilitating the identification of stable QTNs/QTLs and candidate genes associated with FPH, an essential step for molecular breeding of soybean cultivars with improved suitability for mechanized harvesting.

In the present study, we conducted a comprehensive GWAS to dissect the genetic basis of FPH using a diverse natural population consisting of 386 soybean accessions, including wild soybeans, landraces, and released cultivars. Our specific objectives were to (i) evaluate the phenotypic variation and broad-sense heritability of FPH across multiple environments; (ii) identify stable and significant QTNs/QTLs associated with FPH using two GWAS methods, RTM-GWAS [15] (Restricted Two-stage Multi-locus Genome-Wide Association Study) and EMMAX [16] (Efficient Mixed-Model Association eXpedited); (iii) select candidate genes based on sequence variation, gene expression, and functional annotation; and (iv) characterize favorable haplotypes of the candidate gene. These findings provide new insights into the genetic basis of FPH and offer potential molecular targets for soybean breeding programs aimed at improving suitability for mechanized harvesting.

## 2. Results

### 2.1. Phenotypic Variation in First Pod Height in the NJNP-FPH Soybean Population

The NJNP-FPH soybean natural population comprises 386 accessions, including 120 wild soybeans, 145 landraces and 121 released cultivars. FPH was measured as the vertical distance from the cotyledon node to the lowest pod-bearing node [17,18]. The lowest pod-bearing node was defined as the node bearing the first pod on either the main stem or the first effective branch, a branch that bears at least one matured pod at soybean maturity (Figure 1).

FPH was evaluated for 386 accessions across three environments, and best linear unbiased prediction (BLUP) values were calculated from the multi-environment data (Table S1). The average FPH values in 2021XZ, 2021DT, and 2022DT were 7.99 cm, 5.57 cm, and 5.66 cm, respectively, with ranges of 0–33 cm, 0–33 cm, and 0–40 cm, respectively (Table S2, Figure 2). Considerable differences in accumulated growing degree days (GDD) and growing season total precipitation existed among the three experimental environments (Table S3), which may partly explain the observed environmental variation for FPH. The histogram showed a wide distribution of FPH in this population (Figure 2A), with landraces and released cultivars exhibiting significantly greater FPH than wild soybeans (Figure 2B). These results indicate that FPH is a quantitative trait potentially associated with soybean domestication and improvement.

Analysis of variance (ANOVA; Table 1) revealed significant effects of genotype and environment on FPH (*p* < 0.001). Genotypic effects accounted for a substantial proportion of the phenotypic variation in FPH, resulting in a high broad-sense heritability (*H*^2^) of 0.946. In addition, the genotype-by-environment interaction was also significant (*p* < 0.001). The significant genotypic variation observed in the NJNP-FPH population further supports its suitability for subsequent GWAS on FPH.

### 2.2. GWAS on First Pod Height in the NJNP-FPH Soybean Population

Based on 72,679 high-quality single nucleotide polymorphisms (SNPs), GWAS on FPH was performed using two methods, RTM-GWAS and EMMAX. A total of 114 and 24 QTNs/QTLs significantly associated with FPH were identified by RTM-GWAS and EMMAX, respectively (Figure 3 and Figure 4; Table S4). Among these, QTNs/QTLs detected in at least three of the four datasets (three individual environments plus the BLUP dataset) by the same GWAS method (either RTM-GWAS or EMMAX) were considered stable QTNs/QTLs, resulting in four and four stable QTNs/QTL identified by RTM-GWAS and EMMAX, respectively (Table 2). Notably, three stable QTNs/QTL including *FPH_1*, *FPH_2*, and *FPH_3*, were consistently detected by both EMMAX and RTM-GWAS (Table 2).

The physical positions of the seven stable QTNs/QTL were converted to their corresponding coordinates on the Wm82.a2.v1 and ZH13.v2 assemblies (Table S5). The ZH13.v2 coordinates were then used to retrieve previously reported QTLs overlapping with each QTN/QTL in the SoyOmics [19] database (https://ngdc.cncb.ac.cn/soyomics, accessed on 31 July 2026). *FPH_2* and *FPH_4*, located on chromosomes 3 and 9, respectively, overlapped with the previously reported QTLs associated with plant height and internode length, whereas *FPH_5*, *FPH_6*, and *FPH_7* overlapped with a known QTL for lodging (Table 2). *FPH_1* and *FPH_3* are newly identified QTN/QTL for FPH in this study.

### 2.3. Identification of Candidate Genes Associated with First Pod Height

Among the seven stable QTNs/QTL, *FPH_1*, *FPH_3*, *FPH_5, FPH_6*, and *FPH_7* were located in intergenic regions, whereas *FPH_2* and *FPH_4* were located within genes (Table 3). *FPH_2* is located in an intron of *Glyma03G33280* (Wm82.a1.v1.1), which corresponds to *Glyma.03G175700* (Wm82.a2.v1) and *SoyZH13_03G158100* (ZH13.v2). This gene encodes a valine-tRNA ligase, which plays a pivotal role in protein synthesis by catalyzing the specific attachment of valine to its cognate tRNA [20]. *FPH_4* is located within an intron of *Glyma09G10196* (Wm82.a1.v1.1), which corresponds to *Glyma.09G084300* (Wm82.a2.v1) and *SoyZH13_09G078900* (ZH13.v2). This gene encodes a microtubule-associated protein homologous to *Arabidopsis* AIR9 (At2G34680). AIR9 is involved in microtubule organization and division-plane positioning during cell division [21], processes that are essential for cytokinesis and may subsequently influence stem development and elongation. Therefore, *Glyma03G33280* and *Glyma09G10196* were selected for further analysis.

We next examined the expression profiles of *Glyma03G33280* and *Glyma09G10196* in soybean stems across developmental stages from VC to R2 (Figure 5). *Glyma03G33280* (*SoyZH13_03G158100*) exhibited negligible expression (TPM: 1.29–3.17) in soybean stems throughout these developmental stages (Figure 5A). In contrast, *Glyma09G10196* (*SoyZH13_09G078900*) showed consistently high expression levels across these developmental stages (Figure 5B). Based on its localization within the stable QTN *FPH_4*, its strong stem expression, and its annotation as a microtubule-associated protein homologous to Arabidopsis AIR9, *Glyma09G10196* was prioritized as the candidate gene for FPH and was designated *GmAIR9* for subsequent analyses.

### 2.4. Haplotype Analysis of Candidate Gene GmAIR9 Associated with First Pod Height

To further characterize allelic variation in *GmAIR9*, we performed haplotype analysis based on four SNPs identified within *GmAIR9* (Figure 6A). In the soybean NJNP-FPH population, these SNPs defined seven distinct haplotypes, designated H1 to H7. Significant differences in FPH were observed among the major haplotypes (Figure 6B). Accessions carrying H1, H2, and H4 exhibited higher FPH (with mean values of 6.10, 5.50, and 5. 82 cm, respectively) than those carrying H3 (mean: 0.16 cm).

We next examined the distribution of *GmAIR9* haplotypes among wild soybeans, landraces, and released cultivars (Figure 6C). In wild soybeans, H1, H3, and H2 were the three predominant haplotypes, with frequencies of 50.00%, 25.83%, and 20.00%, respectively. Notably, H3 was completely absent in landraces and released cultivars. In contrast, the frequency of H1 increased from 50.00% in wild soybeans to 71.03% in landraces and 83.47% in released cultivars, suggesting that H1 is likely the favorable haplotype that has been preferentially retained during soybean domestication and breeding.

## 3. Discussion

FPH is a pivotal agronomic trait that directly impacts mechanical harvesting efficiency. However, compared to other agronomic traits, the stable QTLs/QTNs and candidate genes underlying FPH remain largely unexplored. In the present study, we evaluated FPH in a diverse natural soybean population across three field environments. Using two GWAS methods, RTM-GWAS and EMMAX, we identified seven stable QTNs/QTL significantly associated with FPH. *GmAIR9*, which encodes a microtubule-associated protein homologous to *Arabidopsis* AIR9 and exhibits high expression in soybean stems, was identified as a candidate gene for FPH meriting further functional investigation. These findings provide valuable insights into the genetic basis of FPH and offer promising candidate loci for future validation, with potential applications in breeding programs to optimize FPH and mechanical harvesting suitability in soybean.

### 3.1. Stable and Significant QTNs/QTL Associated with FPH

As FPH is influenced by environmental conditions, we evaluated FPH across three environments with multiple replications. We also adopted a joint detection strategy using two GWAS methods to reduce the likelihood of false positive associations. Ultimately, seven stable QTNs/QTL significantly associated with FPH were identified, five of which overlapped with previously reported QTLs for plant height, internode length, or lodging. Notably, *FPH_1*, *FPH_2*, and *FPH_3* were independently detected by both EMMAX and RTM-GWAS, further supporting the robustness of our mapping results.

Based on the physical positions of QTNs/QTL identified in this study, the SoyOmics (https://ngdc.cncb.ac.cn/soyomics, accessed on 31 July 2026) database [19] indicated that *FPH_2* co-localized with a previously reported QTL for plant height (*Plant height 17-7*) [23], whereas *FPH_4* overlapped with a previously reported QTL for internode length (*Internode length 2-1*) [24]. These results are consistent with the findings of Kuzbakova et al. [6] that FPH is correlated with plant height and influenced by internode length, further supporting the potential roles of these stable QTNs/QTL in the genetic regulation of FPH in soybean.

### 3.2. Functional Prediction of GmAIR9 in the Regulation of FPH

The stable QTN *FPH_4* is located within *Glyma09G10196* (*GmAIR9*; corresponding to *Glyma.09G084300*/*SoyZH13_09G078900*). *GmAIR9* encodes a microtubule-associated protein. Its Arabidopsis ortholog, *At2G34680* (*AIR9*), has been implicated in cell-plate maturation at the cortical division site [21] and was originally identified as an auxin-inducible gene [25], suggesting a potential connection between *AIR9* and auxin-mediated developmental processes. Auxin is known to regulate stem elongation and branching [26,27], and *GmAIR9* exhibited consistently high expression levels in soybean stems across multiple developmental stages. Based on these observations, we hypothesize that *GmAIR9* may be involved in soybean stem development and FPH regulation, potentially through its role in microtubule organization and cell plate formation during cell division. However, whether *GmAIR9* directly influences cell elongation or functions through an auxin-dependent pathway remains unclear. Further functional validation, including gene knockout and overexpression analyses, will be necessary to determine the precise role of *GmAIR9* in regulating FPH.

## 4. Materials and Methods

### 4.1. Plant Materials and Field Experiments

The soybean natural population (NJNP-FPH) was obtained from the National Center for Soybean Improvement (Nanjing, China). The population comprised 386 accessions, including 120 wild soybeans, 145 landraces and 121 released cultivars.

The soybean accessions of NJNP-FPH were grown in a randomized complete block design (RCBD) under three environments, with three replications in each environment. Field experiments were conducted at two locations, Dangtu Experimental Station (DT) in Anhui Province and Xuzhou Experimental Station (XZ) in Jiangsu Province, across two years. The three environments were designated by year-location as 2021DT, 2021XZ, and 2022DT.

Due to the distinct vine growth habit of wild soybean, which differs fundamentally from the erect growth habit of cultivated soybean, different planting densities and cultivation practices were adopted for wild and cultivated accessions, as described in previous studies [28,29,30]. Wild soybean accessions were planted in 2 m-long rows with a plant spacing of 50 cm and a row spacing of 100 cm. To prevent mutual entanglement, a climbing pole approximately 3 m in height was provided for each wild individual at the seedling stage [28]. Landraces and released cultivars were planted in 2 m-long rows with a plant spacing of 10 cm and a row spacing of 50 cm [29,30]. Standard agronomic management practices were applied throughout the growing season.

At maturity, FPH was measured in centimeters (cm) using a ruler as the vertical distance from the cotyledon node to the lowest pod-bearing node. The lowest pod-bearing node was defined as the node carrying the first (lowest) pod on either the main stem or the first effective branch, whichever was positioned lower [17].

### 4.2. SNP Genotyping and Quality Control

The genotypes of 386 soybean accessions (a subset of the 1024 accessions in our previous study [15]) were called against the Wm82.a1.v1.1 reference genome using the same bioinformatic pipeline described previously [15]. Briefly, SNP genotyping was performed using RAD-seq (Restriction-site Associated DNA sequencing) on an Illumina HiSeq 2000 platform at BGI (Shenzhen, China). Soybean genomic DNA was extracted from bulked leaves at the V4–V5 stage using the CTAB method, and RAD libraries were prepared as described previously [31]. Barcoded adapters (6 nt, ≥2 nt differences) were ligated, and pooled samples were sheared, size-selected (400–700 bp), ligated to a second adapter, PCR-amplified (12 cycles), and sequenced on an Illumina HiSeq 2000 via multiplexed shotgun genotyping [32]. Reads were aligned to the soybean reference genome (*G. max* Wm82.a1.v1.1) [33] using SOAP2 [34], and SNPs were called by RealSFS based on Bayesian estimation of the site frequency [35,36]. Missing SNP genotypes were imputed using FastPHASE [37]. All three tools were run with default parameters. Additional quality control was applied to the SNPs obtained from the previous study [15]; SNPs that were non-polymorphic or had more than 10% missing data were removed. A minor allele frequency (MAF) threshold of ≥ 0.01 was subsequently applied, resulting in a final set of 72,679 high-quality SNPs.

### 4.3. Genome-Wide Association Study on First Pod Height in Soybean

FPH measurements from the three environments (number of phenotyped accessions per environment: 2021XZ: *n* = 374; 2021DT: *n* = 374; 2022DT: *n* = 379; missing phenotypic observations were coded as NA), together with the BLUP values for all 386 accessions in the NJNP-FPH population, were used for GWAS. GWAS was conducted using EMMAX [16] with the mixed linear model (MLM) accounting for population structure and kinship. Principal component analysis (PCA) based on the LD pruned (–indep-pairwise 50 10 0.2) [38] SNP dataset was conducted using GCTA (version 1.92.2beta) [39], and the first ten principal components were included as fixed effects in the MLM (Figure S1), as the scree plot showed that each PC beyond PC10 explained <1.19% of the variance, and these ten PCs together captured most of the genetic variation among accessions. The kinship matrix was calculated using EMMAX based on all high-quality SNPs. Given the total number of SNPs analyzed (n = 72,679), the genome-wide significance threshold was set at *p* ≤ 0.05/72,679 = 6.88 × 10^−7^, corresponding to −log_10_ (*p*) ≥ 6.16, according to the Bonferroni correction. SNPs significantly associated with FPH were designated as FPH QTNs.

RTM-GWAS was additionally used to identify SNPs or SNP linkage disequilibrium block (SNPLDB) markers potentially associated with FPH [40]. In the first stage, a single-locus association test was performed to eliminate redundant SNPs/SNPLDBs using a nominal significance threshold of *p* ≤ 0.05. In the second stage, forward selection and backward elimination were performed using stepwise regression under a multilocus model to identify genome-wide QTNs from the reduced marker set, using an experiment-wide significance threshold of *p* ≤ 0.05 [41].

GWAS results were visualized using scripts from the R package CMplot [42] (https://cran.r-project.org/web/packages/CMplot/index.html, accessed on 20 July 2026).

### 4.4. Candidate Gene Identification

To identify potential candidate genes for FPH, the physical positions of the seven stable and significant QTNs/QTL (*FPH_1* to *FPH_7*) were examined based on the soybean Wm82.a1.v1.1 reference genome [33]. For comprehensive and accurate gene annotation, the physical coordinates of the significant QTNs/QTL were converted to their corresponding coordinates on the Wm82.a2.v1 and ZH13.v2 assemblies (Table S5) using the SoyOD [22] database (https://bis.zju.edu.cn/soyod/, accessed on 31 July 2026).

Functional annotations of genes located within the QTN/QTL intervals were retrieved from the SoyOmics [19] (https://ngdc.cncb.ac.cn/soyomics/, accessed on 31 July 2026) database. To further narrow down the list of candidate genes, the expression patterns of the selected genes in soybean stems were investigated using transcriptome datasets available in SoyOmics [19] across different developmental stages, from the vegetative cotyledon (VC) stage to the reproductive R2 stage. A heatmap was generated using the SoyOmics [19] database to visualize the expression patterns of these genes.

### 4.5. Haplotype Analysis of GmAIR9 in the NJNP-FPH Soybean Population

To investigate allelic variation in the candidate gene *GmAIR9*, its genomic region, including the 2 kb promoter region, was analyzed in the NJNP-FPH soybean population. SNPs with less than 10% missing data and a minor allele frequency (MAF) > 1% were used for haplotype analysis. Based on their allelic combinations, the accessions were classified into distinct haplotypes, designated H1–H7. The frequencies of these haplotypes were then calculated across the three subpopulations: wild soybeans, landraces, and released cultivars.

### 4.6. Statistical Analyses

Mean FPH values were calculated using Microsoft Excel 2019. Phenotypic variation and descriptive statistics for FPH were analyzed using the MEANS and PROC GLM procedures in SAS 9.2 (SAS Institute Inc., Cary, NC, USA). BLUP values for FPH were calculated for each accession using the lme4 package [43,44] in R (version R-4.5.1; http://www.R-project.org/). Broad-sense heritability was estimated across multiple environments using the following equation:$\;H^{2}=\sigma_{g}^{2}/(\sigma_{g}^{2}+\sigma_{ge}^{2}/n+\sigma_{e}^{2}/nr)\times 100\%$; where $\sigma_{g}^{2}$, $\sigma_{ge}^{2}$ and $\sigma_{e}^{2}$ represent the genotypic variance, genotype-by-environment interaction variance, and residual error variance, respectively; n is the number of environments, and r is the number of replications [29,45]. For comparisons among multiple groups, one-way analysis of variance (ANOVA) followed by Tukey’s multiple-comparison test was performed using GraphPad Prism (version 10.4.1).

## 5. Conclusions

This study demonstrated substantial genetic variation in first pod height (FPH) within the natural soybean population. Using GWAS, we identified seven stable QTNs/QTL significantly associated with soybean FPH. Among these loci, *GmAIR9*, which encodes a microtubule-associated protein, was prioritized as a candidate gene meriting further functional investigation, based on its genomic location, annotation, and strong expression in soybean stems. Haplotype analysis further revealed that the *GmAIR9* H1 haplotype was associated with higher FPH, and this H1 haplotype was progressively enriched from wild soybeans to landraces and improved cultivars. Collectively, this study provides useful genetic resources and potential molecular targets for improving FPH and optimizing soybean plant architecture for mechanized harvesting.

## Figures and Tables

**Figure 1:**
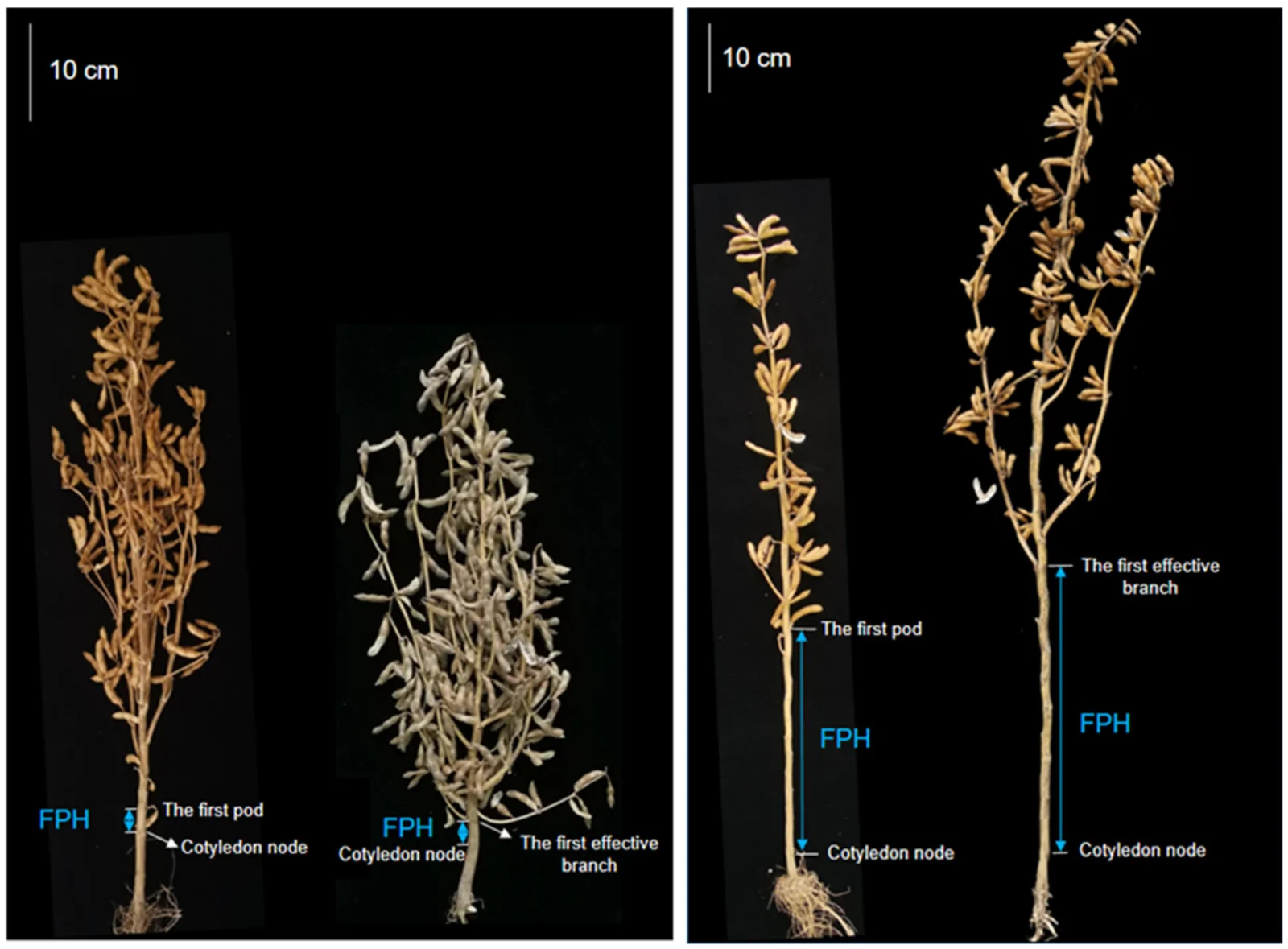
Schematic diagram illustrating the measurement of first pod height (FPH). FPH was defined as the vertical distance from the cotyledon node to the lowest pod-bearing node. The lowest pod-bearing node was identified as the node with either the first pod on the main stem or on the first effective branch, a branch that bears at least one mature pod at soybean maturity.

**Figure 2:**
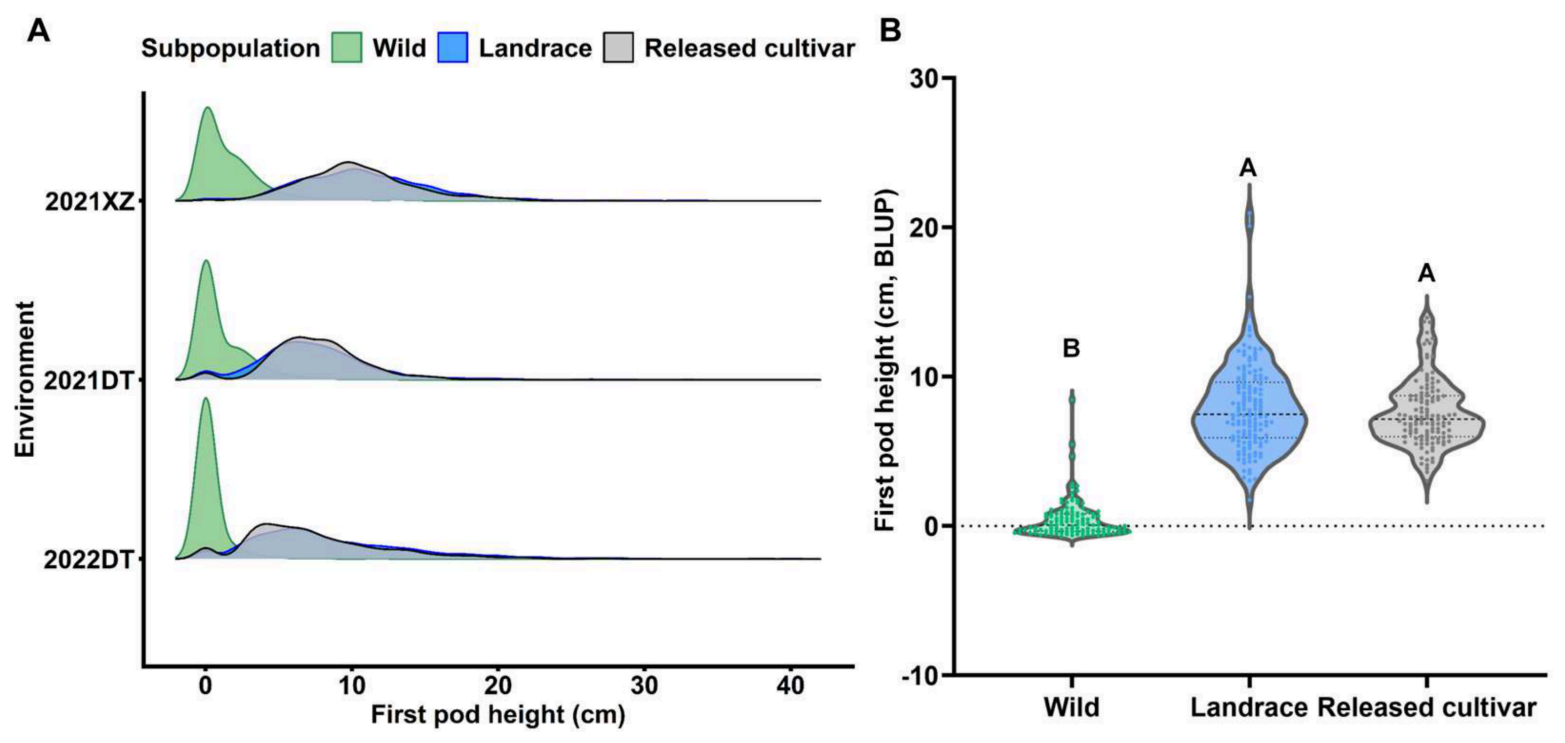
Phenotypic variation in first pod height (FPH) in the NJNP-FPH soybean population. (**A**) Distribution of FPH under three environments in the NJNP population. The years 2021 and 2022 represent the years of phenotypic evaluation; XZ and DT denote the evaluation locations Xuzhou and Dangtu, respectively. (**B**) Comparison of FPH among the three subpopulations: wild soybeans, landraces, and released cultivars. The *y*-axis represents the BLUP (best linear unbiased prediction) values of FPH across three environments. Different uppercase letters above the boxes indicate significant differences determined by one-way analysis of variance (ANOVA) followed by Tukey’s multiple comparison test (α = 0.001).

**Figure 3:**
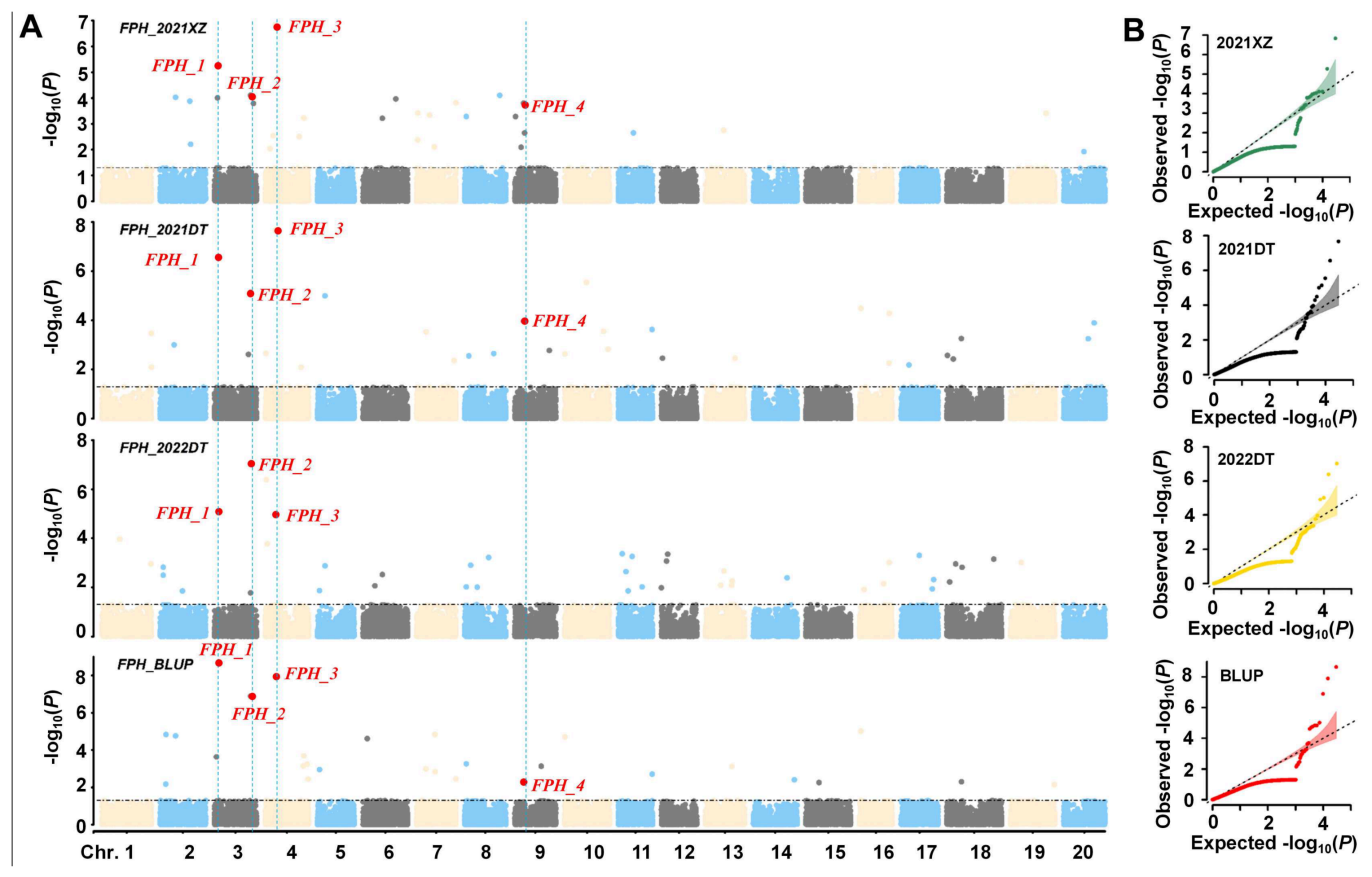
GWAS of first pod height (FPH) in the NJNP-FPH population of 386 soybean accessions using RTM-GWAS. (**A**) Manhattan plots showing the genome-wide association signals for FPH by RTM-GWAS. The horizontal black dotted line represents the genome-wide significance threshold of *p* = 0.05, corresponding to −log_10_(*p*) = 1.30. Red points and labels denote stable QTNs/QTL detected in at least three analyses. Blue vertical dashed lines highlight the significant QTNs/QTL that are stable and co-localized across different environments. (**B**) Quantile–quantile plots for RTM-GWAS results. The years 2021 and 2022 represent the years of phenotypic evaluation; XZ and DT denote the evaluation locations Xuzhou and Dangtu, respectively.

**Figure 4:**
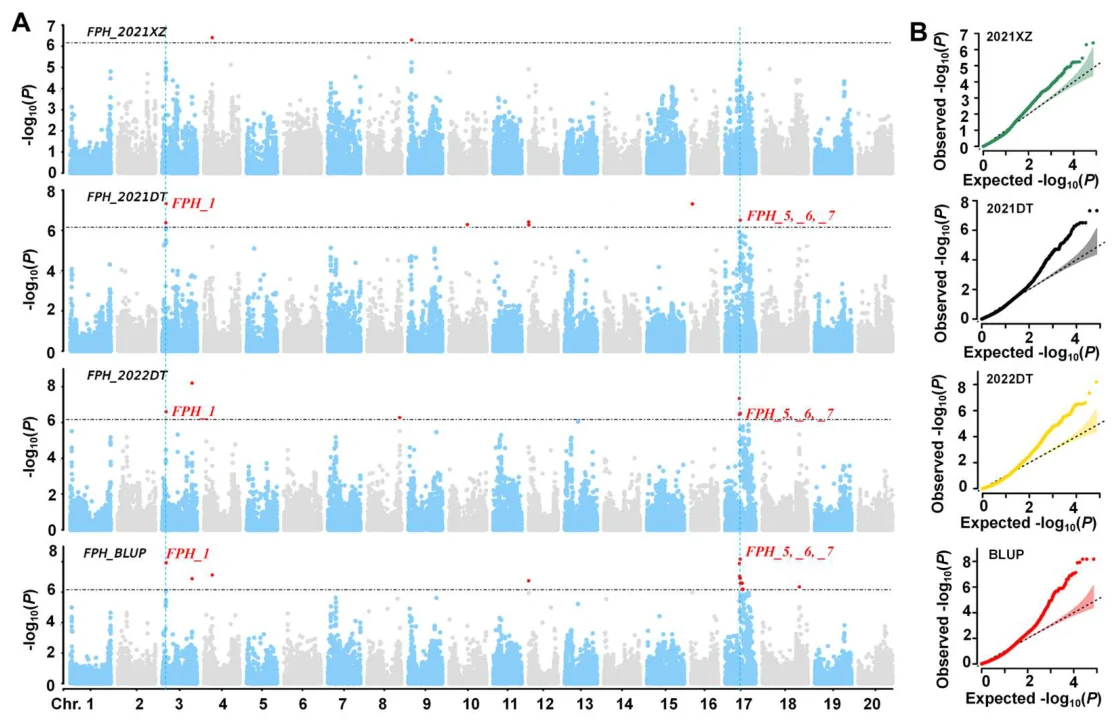
GWAS of first pod height (FPH) in the NJNP-FPH population of 386 soybean accessions using EMMAX. (**A**) Manhattan plots showing the genome-wide association signals for FPH identified by EMMAX. The horizontal black dashed line indicates the significance threshold of *p* = 0.05/*n*, corresponding to −log_10_(*p*) = 6.16, where *n* is the total number of SNPs. Red points and labels denote stable QTNs detected in at least three analyses. Blue vertical dashed lines highlight the significant QTNs/QTL that are stable and co-localized across different environments. (**B**) Quantile–quantile plots of the EMMAX results. The years 2021 and 2022 represent the years of phenotypic evaluation; XZ and DT denote the evaluation locations Xuzhou and Dangtu, respectively.

**Figure 5:**
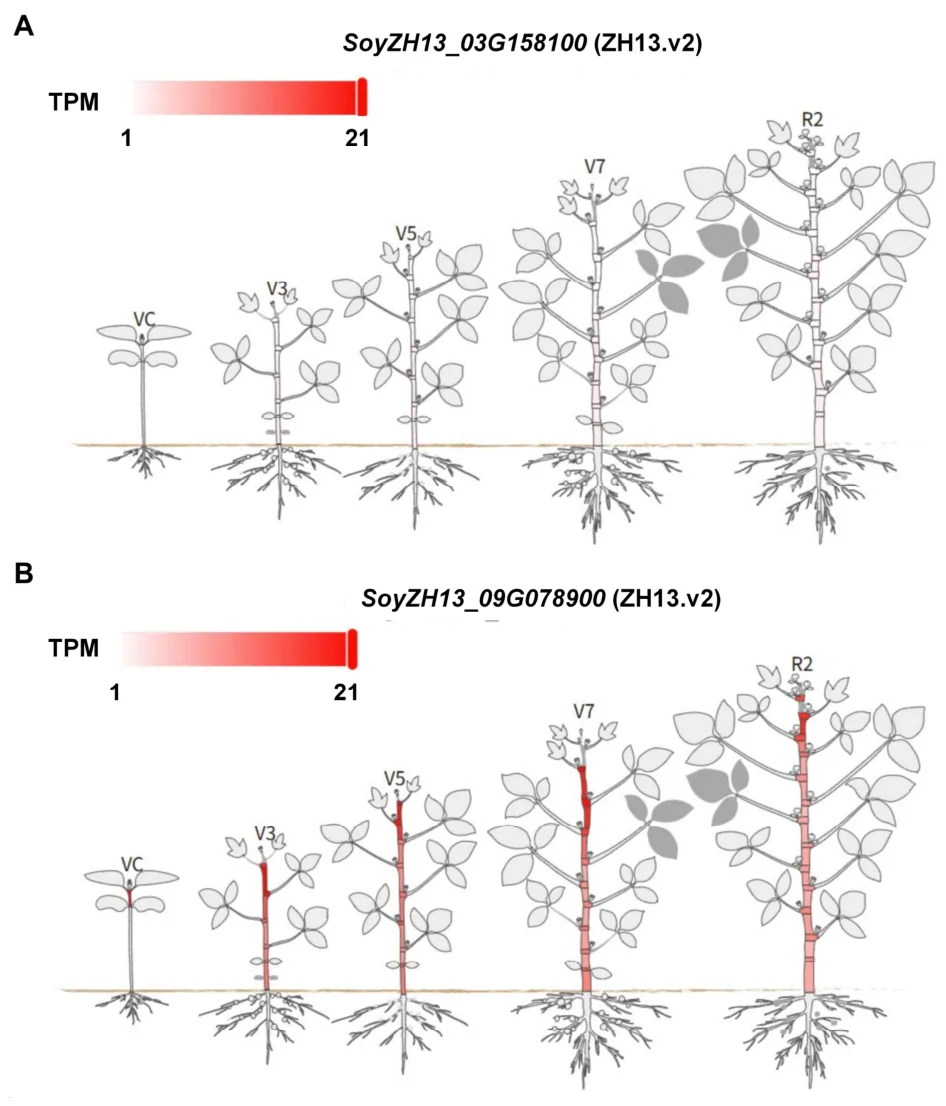
(**A**,**B**) Expression patterns of *Glyma03G33280* (*SoyZH13_03G158100*) and *GmAIR9* (*SoyZH13_09G078900*) in soybean stems at the VC, V3, V5, V7, and R2 developmental stages, based on transcriptome data from the SoyOmics [19] database (https://ngdc.cncb.ac.cn/soyomics, accessed on 31 July 2026). TPM, transcripts per million.

**Figure 6:**
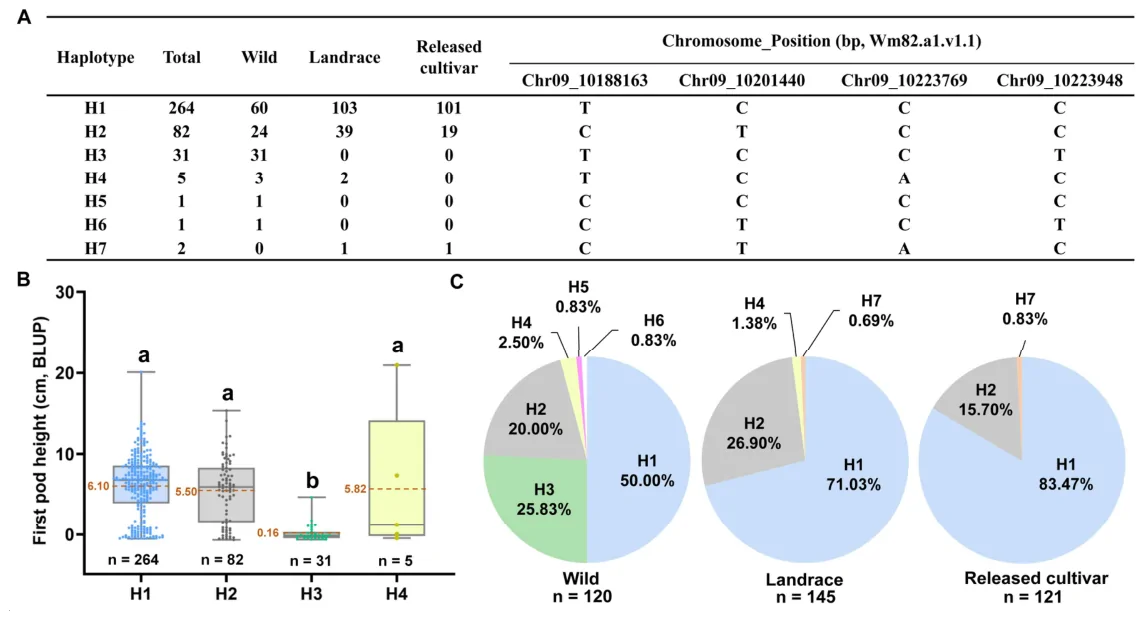
Haplotype analysis of *GmAIR9* in the NJNP-FPH soybean population. (**A**) SNP and haplotypes of *GmAIR9* in *Glycine soja* (wild soybeans) and *Glycine max* (landraces and released cultivars). Four polymorphic SNPs within the intron of *GmAIR9* defined seven haplotypes. (**B**) Allelic effects of the four major *GmAIR9* haplotypes on first pod height (FPH). The *Y*-axis represents the best linear unbiased prediction (BLUP) values of FPH across three environments. Only haplotypes with at least five accessions are shown. Different lowercase letters above the boxes indicate significant differences determined by one-way analysis of variance (ANOVA) followed by Tukey’s multiple comparison test (α = 0.05). The mean values are shown as red dashed lines in the figure. (**C**) Frequencies of *GmAIR9* haplotypes in the three subpopulations: wild soybeans, landraces, and released cultivars. Percentages for wild soybeans may not sum to 100% due to rounding.

**Table 1:** Analysis of variance for first pod height in the NJNP-FPH soybean population.

Variation	DF	SS	MS	*F* Value	*p* Value
Genotype	385	56,000.71	145.46	36.21	<0.0001
Environment	2	3447.96	1723.98	429.15	<0.0001
Replications (Environment)	6	544.38	90.73	22.59	<0.0001
Genotype × Environment	739	6209.57	8.40	2.09	<0.0001
Error	2015	8094.57	4.02		

**Table 2:** Seven stable QTNs/QTL associated with first pod height in soybean.

QTN/QTL	Method	SNP/Block	Association *p* Value				Reported QTLs ^2^ Related to FPH
		(Chr._Position) ^1^	2021XZ	2021DT	2022DT	BLUP	
*FPH_1*	RTM-GWAS	Chr03_5187477	5.42 × 10^−6^	2.76 × 10^−7^	9.40 × 10^−6^	2.27 × 10^−9^	Newly identified
	EMMAX			4.65 × 10^−8^	2.49 × 10^−7^	1.19 × 10^−8^	
*FPH_2*	RTM-GWAS	Chr03_40913591	7.81 × 10^−5^	7.22 × 10^−6^	9.43 × 10^−8^	1.28 × 10^−7^	*Plant height 17-7*
	EMMAX				6.26 × 10^−9^	1.31 × 10^−7^	
*FPH_3*	RTM-GWAS	Block_4_11374739_11375027	1.48 × 10^−7^	2.17 × 10^−8^	1.23 × 10^−5^	1.27 × 10^−8^	Newly identified
	EMMAX	Chr04_11374739	3.88 × 10^−7^			7.45 × 10^−8^	
*FPH_4*	RTM-GWAS	Chr09_10223769	1.59 × 10^−4^	1.08 × 10^−4^		5.62 × 10^−3^	*Internode length 2-1*
*FPH_5*	EMMAX	Chr17_20689473		3.08 × 10^−7^	3.10 × 10^−7^	6.76 × 10^−9^	*Lodging 20-1*
*FPH_6*	EMMAX	Chr17_20846301		3.08 × 10^−7^	3.10 × 10^−7^	6.76 × 10^−9^	*Lodging 20-1*
*FPH_7*	EMMAX	Chr17_20849092		3.08 × 10^−7^	3.10 × 10^−7^	6.76 × 10^−9^	*Lodging 20-1*

^1^ Chr._Position refers to the physical position (bp) based on the Wm82.a1.v1.1 reference genome; ^2^ Previously reported soybean QTLs were retrieved from the SoyOmics [19] database (https://ngdc.cncb.ac.cn/soyomics, accessed on 27 July 2026), using the ZH13.v2 physical coordinates (Table S5) of each identified QTN/QTL. A QTN/QTL was considered newly identified when its physical interval did not overlap with any previously reported QTL related to first-pod height (FPH), plant height, internode length, or lodging.

**Table 3:** Candidate genes associated with first pod height of stable QTNs.

QTN	SNP (Chr._Pos.) ^1^	Position in Gene	Wm82.a1.v1.1	Wm82.a2.v1	ZH13.v2	Functional Description ^2^
*FPH_2*	Chr03_40913591	Intron	*Glyma03G33280*	*Glyma.03G175700*	*SoyZH13_03G158100*	Valine-tRNA ligase
*FPH_4*	Chr09_10223769	Intron	*Glyma09G10196*	*Glyma.09G084300*	*SoyZH13_09G078900*	microtubule-associated protein AIR9

^1^ Chr._Position refers to the physical position (bp) based on the Wm82.a1.v1.1 reference genome; ^2^ Functional description was obtained from the SoyOmics [19] (https://ngdc.cncb.ac.cn/soyomics, accessed on 27 July 2026) database. Gene model identifiers across different soybean reference genomes were converted using SoyBase [10] (https://www.soybase.org, accessed on 27 July 2026) and SoyOD [22] (https://bis.zju.edu.cn/soyod/, accessed on 27 July 2026).

## Data Availability

The data presented in this study are available in the Supplementary Materials. Further inquiries may be directed to the corresponding author.

## Supplementary Materials

The following supporting information can be downloaded at: https://www.mdpi.com/article/10.3390/plants15192937/s1, Figure S1. Principal component analysis of 386 soybean accessions, (A) The three-dimensional plot shows the distribution of wild soybeans, landraces, and released cultivars along the first three principal components (PC1, PC2, and PC3). (B) Scree plot showing the percentage of variance explained (PVE) by each principal component (PC). Table S1. First pod height and BLUP values for 386 soybean accessions in the NJNP-FPH population across three environments. Table S2. Descriptive statistics of first pod height in the NJNP-FPH soybean population. Table S3. Growing-season meteorological conditions across three environments. Table S4. Significant QTNs/QTLs associated with first pod height identified in this study. Table S5. Physical positions of stable QTNs/QTLs across three soybean reference genome assemblies. References [46,47,48] are cited in the Supplementary Materials.

## Abbreviations

The following abbreviations are used in this manuscript:

FPH	First pod height
BLUP	Best linear unbiased prediction
SNP	Single nucleotide polymorphism
GWAS	Genome-wide association study
QTN	Quantitative trait nucleotide
QTL	Quantitative trait locus
TPM	Transcripts per million
