# Supplementary material for: Genome-Wide Association Study and Candidate Gene Mining for First Pod Height in Soybean
Source: Plants (Basel). 2026 Sep 25;15(19):2937. doi: 10.3390/plants15192937 (PMC13667089; doi:10.3390/plants15192937)
Supplement: Supplementary file 1 [file plants-15-02937-s001.zip › Supplementary Figure.pdf]

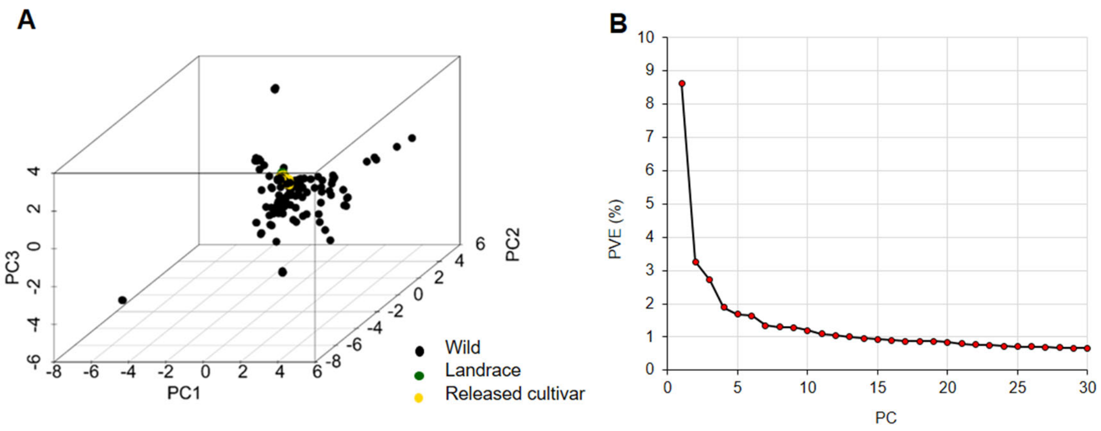

Figure S1. Principal component analysis of 386 soybean accessions. (**A**) The three-dimensional plot shows the distribution of wild soybeans, landraces, and released cultivars along the first three principal components (PC1, PC2, and PC3). (**B**) Scree plot showing the percentage of variance explained (PVE) by each principal component (PC).
